# Tamarixinin A Alleviates Joint Destruction of Rheumatoid Arthritis by Blockade of MAPK and NF-κB Activation

**DOI:** 10.3389/fphar.2017.00538

**Published:** 2017-08-15

**Authors:** Yuanyuan Zhuang, Jiabao Liu, Pei Ma, Jinye Bai, Yasi Ding, Hui Yang, Yannan Fan, Mingbao Lin, Shuai Li, Qi Hou

**Affiliations:** State Key Laboratory of Bioactive Substances and Functions of Natural Medicines, Institute of Materia Medica, Chinese Academy of Medical Sciences and Peking Union Medical College Beijing, China

**Keywords:** tamarixinin A, rheumatoid arthritis (RA), mouse, rat, MAPK, NF-κB

## Abstract

**Background:** Tamarixinin A, a natural tannin isolated from *Myricaria bracteata*, has been confirmed to have moderate anti-inflammatory effects *in vitro* and *in vivo*. However, how it effects rheumatoid arthritis (RA) is still unknown. Therefore, the aim of this study is to investigate the therapeutic effects of tamarixinin A on experimental RA, and explore the underlying mechanism.

**Methods:** The anti-arthritic effects of tamarixinin A were evaluated on collagen-induced arthritis (CIA) mice and adjuvant-induced arthritis (AIA) rats. The hind paw thickness, inflammatory cytokine levels in serum, and histopathological assessments were determined. The arthritis score was evaluated. Activation of p38 and p65 in AIA rats was also determined. The anti-inflammatory effect *in vitro* was also tested in LPS induced macrophages, and its related anti-inflammatory signaling pathways were explored.

**Results:** Treatment with tamarixinin A significantly suppressed the progression and development of RA in CIA mice and AIA rats. Both in CIA mice and AIA rats, arthritis scores decreased, paw swelling and thickness were reduced, and joint destruction was alleviated. In AIA rats, tamarixinin A significantly inhibited the expression of p38, p-p38 and p65. In addition, tamarixinin A inhibited the production of pro-inflammatory mediators, the phosphorylation of p38, ERK, JNK and p65, as well as the nuclear translocation of p38 in LPS- induced macrophages.

**Conclusion:** Tamarixinin A is a potential effective candidate compound for human RA treatment, which executes anti-arthritic effects potentially through down-regulating MAPK and NF-κB signal pathway activation.

## Introduction

Rheumatoid arthritis (RA) is a systemic inflammatory autoimmune disease with a prevalence of about 1% in the general population, which principally attacks flexible (synovial) joints with synovial hyperplasia and joint destruction, and eventually results in deformity, loss of function, and reduced quality of life (Smolen et al., 2016). Corticosteroids, non-steroidal anti-inflammatory drugs (NSAIDs), and disease-modifying antirheumatic drugs (sDMARDs and bDMARDs) are the main drugs used for RA management in the clinic (Roubille et al., 2015). However, 30–50% of patients do not effectively respond to these therapies due to various adverse effects, including gastrointestinal lesions, cardiovascular complications, and hepatotoxicity (Mócsai et al., 2014). The limited effectiveness and adverse effects of current therapies highlight the urgent need for new anti-inflammatory drugs for RA treatment.

**FIGURE 1:**
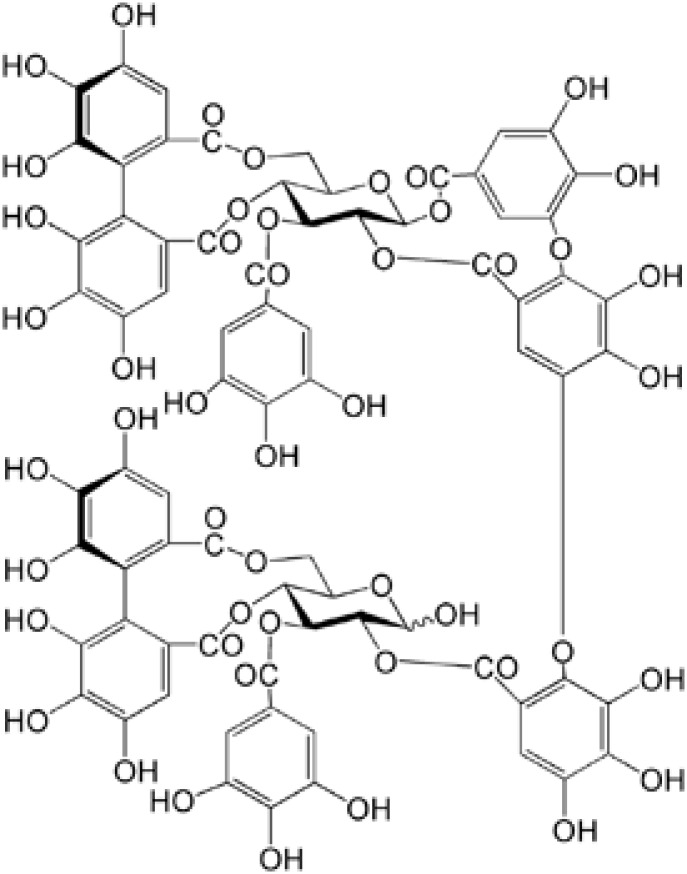
The chemical structure of tamarixinin A.

Medicinal plants are important sources of novel therapeutic candidates for RA treatment. Natural products capable of anti-inflammatory effects and reducing the toxicity of pharmacological agents have led to the discovery of potential novel alternative therapeutic agents (Khanna et al., 2007; Doss et al., 2016). *Myricaria bracteata* Royle (Tamaricaceae), a plant widely distributed in Asia and Europe, has been used as a treatment for inflammatory disease, including rheumatism and arthritis (Liu et al., 2015). Literature concerning the isolation of phytochemical constituents from Tamaricaceae is extensive. Our previous study showed that tamarixinin A (**Figure 1**), a major hydrolyzable tannin isolated from the hydrophilic portion of Tamaricaceae, has a dose dependent anti-inflammatory effect in the inhibition of ear swelling in croton oil-induced ear edema in mice, and a 46% inhibitation rate in collagen-induced paw edema with 20 mg/kg Tamaricaceae treatment in DBA/1 mice (Liu et al., 2015). However, the exact effect of tamarixinin A in arthritis has not been confirmed, and its anti-arthritis molecular mechanisms have not been studied.

In RA, macrophages are the key effector cells in the acute and chronic phases by secreting pro-inflammatory cytokines and mediators that drive angiogenesis, infiltration by immune cells, and cartilage and bone destruction (Clavel et al., 2016). Macrophage number increase in the inflamed synovium due to expansion of resident macrophages and infiltration by circulating monocytes (Hamilton and Tak, 2009). Recent studies suggest that nuclear factor κB (NF-κB) and mitogen-activated protein kinases (MAPKs) are highly activated in macrophages and involved in the pathogenesis of RA (IB, 2005; Brennan et al., 2006; Zhang et al., 2015). Blocking intracellular NF-κB/IKK2 and p38 MAPK signaling pathways to find a new generation of anti- inflammatory drugs for RA has been a focus for many groups (Szekanecz and Koch, 2007; Drexler et al., 2008; Clark and Dean, 2012). NF-κB and MAPK signaling pathways engage macrophages in the release of cytokines, such as TNF-α and interleukin-1, which lead to bone destruction and synovitis (Szekanecz and Koch, 2007; Drexler et al., 2008). These signaling pathways also promote migration of macrophages and cause synovitis via activating 2 key macrophage-derived chemokines (MCP-1 and MIP-1) (Zheng et al., 2009).

In this study, different animal models of RA have been used to determine the effectiveness of tamarixinin A. Adjuvant-induced arthritis (AIA) was used to investigate an experimental autoimmune disease with several features of human RA, while collagen-induced arthritis (CIA) was used for studying disease pathogenesis and validation of potential therapeutic targets. Furthermore, the underlying anti-arthritic mechanisms of tamarixinin A in NF-κB and MAPK pathway were investigated in AIA rats and cultured macrophages *in vitro.*

## Materials and Methods

### Animals

Sixty male DBA/1 mice (weighting 18–22 g) were purchased from Beijing HFK Bioscience Co., Ltd (Beijing, China), and 72 male Wistar rats (weighting 180–220 g) were purchased from Vital River Laboratory Animal Technology Co. Ltd (Beijing, China). Animals were housed in a room on a 12-h light/dark cycle under specific pathogen-free conditions, maintaining the temperature (22–26°C) and relative humidity (55–65%). Standard laboratory chow and water were provided *ad libitum*. All animal experimental procedures were approved by Experimental Animal Care and Use Committee of the Institute of Materia Medica, Chinese Academy of Medical Sciences & Peking Union Medical College (No. 20140912).

### Collagen-Induced Arthritis (CIA) in DBA/1 Mice

**FIGURE 2:**
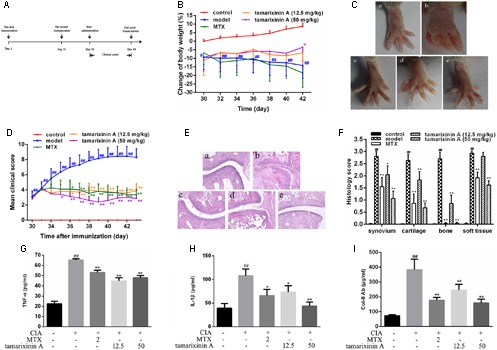
Effects of tamarixinin A on DBA/1 mice with CIA. **(A)** Timeline for the development and treatment process of CIA. **(B)** Body weight changes of mice during drug administration, the changes in body weights were shown as % loss of body weight of control group on day 30. **(C)** Representative photographs of hind paws on day 43 (*n* = 12). **(D)** Clinical scores of CIA mice (*n* = 12). **(E)** Representative images of histological examination of hind paw joints on day 43 (200×), (a) control mice, (b) CIA model mice, (c) CIA mice treated with MTX, (d) CIA mice treated with 12.5 mg/kg of tamarixinin A, (e) CIA mice treated with 50 mg/kg of tamarixinin A. **(F)** Semi-quantitative scores of histological examination (*n* = 3). **(G)** The level of TNF-α production in plasma (*n* = 12). **(H)** The level of IL-β production in plasma (*n* = 12). **(I)** The level of collegan II autoantibody in plasma (*n* = 12). Data are presented as means ± SD. ^#^*P* < 0.05 and ^##^*P* < 0.01 versus control group, ^∗^*P* < 0.05 and ^∗∗^*P* < 0.01 versus CIA model group.

The timeline for the development of CIA and treatment was shown in **Figure 2A**. Native chicken collagen type II (CII; 2 mg/ml in 0.1 M acetic acid; Sigma, St. Louis, MO, United States) was vortexed at 4°C overnight, and was emulsified in an equal volume of Freund’s complete adjuvant (CFA, Sigma) to a final concentration of 1 mg/ml. Each mouse received two intradermal injections 0.1 ml of CII emulsion at two separated sites tail base on day 1 and day 21, respectively. Mice receiving 0.05 M acetic acid were included as blank control. On day 30, immunized mice were randomly divided into four groups (*n* = 12) according to its clinical score, and receiving tamarixinin A (12.5 or 50 mg/kg per day through s.c. on the back), methotrexate (MTX, 2 mg/kg per day through gastric gavage) or vehicle, starting from day 30 to day 44 after the first CII injection. Arthritis scoring was performed on alternate days after the initiation of tamarixinin A / methotrexate treatment. On day 44, the mice were sacrificed by euthanasia and blood was collected for serum separation. The hind limbs were dissected and stored in 10% formalin for histopathological assessment.

### Complete Freund’s Adjuvant-induced Arthritis in Wistar Rats

**FIGURE 3:**
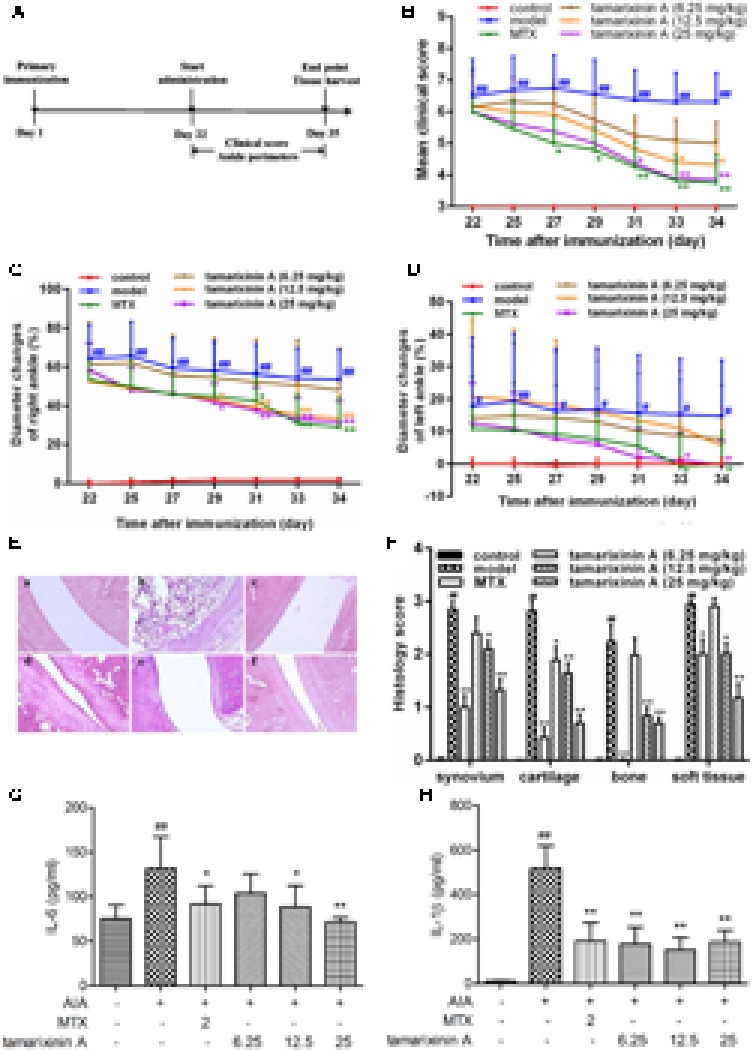
Effects of tamarixinin A on Wistar rats with AIA. **(A)** Timeline for the development and treatment process of AIA. **(B)** Clinical scores of AIA rats (*n* = 12). **(C)** The diameter changes of right hind paws with primary swelling (*n* = 12), which were calculated by comparision with mean ankle diameter of control group on day 22. **(D)** The diameter changes of left hind paws with secondary swelling (*n* = 12), which were calculated by comparision with mean ankle diameter of control group on day 22. **(E)** Representative images of histological examination of the right hind paw joints (200×), (a) control, (b) AIA model, (c) AIA rats treated with MTX, (d) AIA rats treated with 6.25 mg/kg of tamarixinin A, (e) AIA rats treated with 12.5 mg/kg of tamarixinin A. (f) AIA rats treated with 25 mg/kg of tamarixinin A. **(F)** Semi-quantitative scores of histological examination (*n* = 3). **(G)** The level of IL-6 production in joint space flushing fluid (*n* = 12). **(H)** The level of IL-β production in joint space flushing fluid (*n* = 12). Data are presented as means ± SD. ^#^*P* < 0.05 and ^##^*P* < 0.01 versus control group, ^∗^*P* < 0.05 and ^∗∗^*P* < 0.01 versus AIA model group.

**FIGURE 4:**
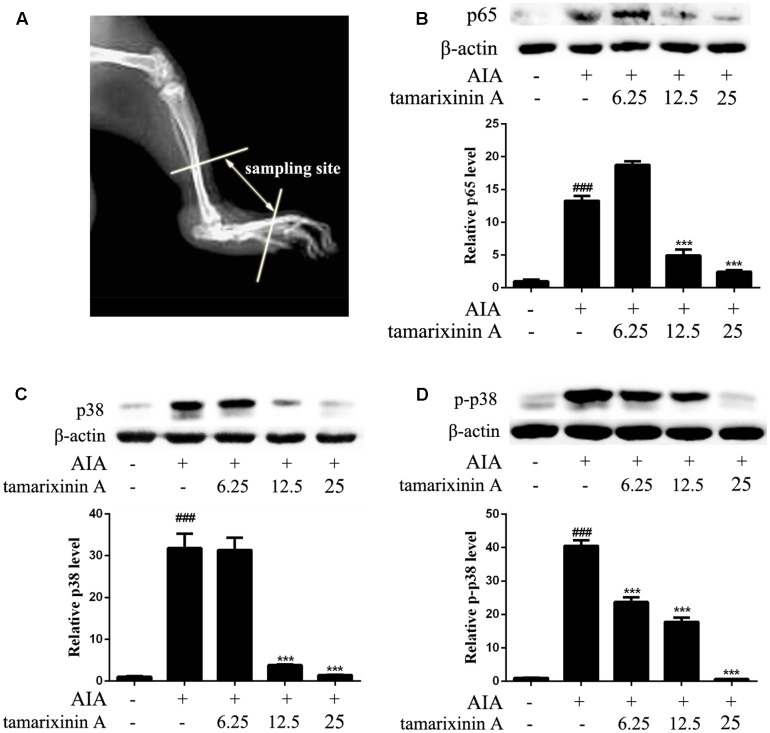
Effects of tamarixinin A on the expression of p38, p-p38, and p65 in AIA rat. **(A)** Sampling site in ankle joints of AIA rats for western blotting analysis. **(B)** The expression level of p65. **(C)** The expression level of p38. **(D)** The phosphorylation levels of p38. Data are presented as means ± SD, *n* = 3. ^###^*P* < 0.001 versus blank control group, ^∗∗∗^*P* < 0.001 versus AIA model group.

The timeline for the development of AIA and treatment was shown in **Figure 3A**. AIA was induced by a single intradermal injection of 0.1 ml CFA (containing 10 mg of heat-killed *Mycobacterium tuberculosis* in 1 ml of paraffin oil) into the footpad of the right hind paw on day 1. Rats (*n* = 12) receiving a single dose of 0.1 ml of paraffin oil – water emulsion without *Mycobacterium tuberculosis* were included as blank control. On day 22, immunized rats were randomly divided into five groups according its clinical score, received tamarixinin A (6.25, 12.5, or 25 mg/kg per day through s.c. on the back), methotrexate (2 mg/kg per day through gastric gavage) or vehicle (equally volumes per day through s.c. on the back), starting from day 22 to day 35. Arthritis scoring, and arthritis diameter (cm) measuring by one tape measure method were performed on alternate days after the initiation of tamarixinin A/methotrexate treatment. On day 35, rats were sacrificed by euthanasia, the hind limbs were dissected, three of them were randomly chosen from each group and stored in 10% formalin for histopathological assessment, another of them were trimmed of skin, cut out ankle joints (sampling sites shown in **Figure 4A**) and homogenized in 3 ml of RIPA lysis buffer containing protease inhibitor cocktail (Roche Diagnostics, Mannheim, Germany) using a tissue homogenizer. The homogenates were incubated for 2 h on ice to obtain full lysis, and then centrifuged at 15,000 ×*g* for 15 min at 4°C, the supernatants were transferred in aliquots to new tubes and stored at -70°C before western blotting analysis.

### Arthritis Scoring

The arthritis scoring were defined by using a semi-quantitative clinical scoring system for each paw, in which 0 = normal, 1 = definite erythema and swelling of the ankle or one digits, 2 = two or more joints involved or mild erythema and swelling of the entire paw, 3 = erythema and swelling extending from the ankle to the metatarsal joints of the entire paw and all digits, and 4 = ankylosing deformity with joint severe erythema and swelling. The arthritis score for each mouse was the sum of all paw scores (totaling a maximum score of 16 per mouse or rat).

### Histological Assessment

The dissected mice or rat left hind paw joints were fixed in 10% formalin solution and decalcified using 12% disodium EDTA. After dehydration, specimens were then paraffin embedded, sectioned (5 μm thickness) for hematoxylin and eosin (H&E) staining, and observed under a light microscope. Histopathological scoring was performed in a blinded manner by experienced pathologists, and classified into four grades for inflammatory activity, synovial hyperplasia, cartilage degradation, and bone erosion, in which 0 = normal, 1 = slight, 2 = moderate, 3 = severe.

### Murine Macrophage Isolation and Culture

Peritoneal macrophages were isolated from C57BL/6 mice 3 days after intraperitoneal injection with 1.2 ml of 4% sterile thioglycolate broth. Then cells were seeded onto 96-well plates at a density of 2 × 10^5^ cells per well in RPMI 1640 medium with 5% fetal bovine serum for 2 h to allow the macrophages to adhere to the plates. The adherent macrophages were cultured in fresh medium containing tamarixinin A (5, 10, and 20 μM, respectively) for 2 h, then incubated with 1 μg/ml of LPS for 24 h. The culture supernatants were collected for further determination of nitric oxide (NO) and cytokines.

For western blotting analysis, the macrophages were plated in culture dishes (60 mm in diameter) at a density of 8 × 10^6^ cells, incubated with tamarixinin A (5, 10, and 20 μM, respectively) for 2 h, and then cultured with 1 μg/ml of LPS for 15 min. The cells were washed with PBS three times, and lysed with RIPA lysis buffer containing protease inhibitor cocktail (Roche Diagnostics, Mannheim, Germany) to extract the total cellular protein.

### Cytokines Measurement by ELISA

The cytokine levels in the culture supernatants of macrophages and serum of experimental mice or rats were determined by using murine or rat specific ELISA kits (Biolegend, San Diego, CA, United States) for TNF-α, IL-1β and IL-6 according to the manufacturer’s instructions. Serum levels of anti-mouse CII antibody in CIA mice were monitored by ELISA (Senbeijia Biological Technology Co., Ltd., Nanjing, China) as the manufacturer’s recommendation.

### Measurement of Nitric Oxide

Culture supernatants of macrophages were collected and NO levels were determined by using Griess reagent (1% sulfanilamide, 0.1% *N*-1-naphthylenediamine dihydrochloride, and 2.5% phosphoric acid). The absorbance was measured at 570 nm with a microplate reader.

### Western Blotting Analysis

Analysis of the proteins extracted from macrophages or ankle joints by Western blot was performed using standard methods. Equivalent amounts of protein from each sample were separated in 10% SDS-polyacrylamide gel and blotted onto a PVDF membrane. The membrane was then blocked with 5% milk (BD, Sparks, MD, United States) and incubated with antibodies against p38, p65, p-p38, p-ERK, p-JNK1/2, p-p65, iNOS, and β-actin (Cell Signaling Technology, Danvers, MA, United States) overnight, and then was hybridized with HRP-conjugated secondary antibody for 1 h. The immunoreactive bands were visualized using a ECL system (CLINX, Shanhai, China). The relative intensities of bands were quantified using Image J.

### Immunofluorescence Staining

Macrophages were plated in 96-well plates at a density of 5 × 10^4^ cells, treated with tamarixinin A (5, 10, and 20 μM, respectively) for 2 h, and then incubated with 1 μg/ml of LPS for 15 min. The cells were then washed and fixed with 4% paraformaldehyde for 30 min. Cells were incubated sequentially with 0.1% triton X-100 for 15 min and blocked with 5% BSA for 30 min. Next, cells were incubated with primary anti-p-p38 antibody (1:400) overnight at 4°C, and TRITC-conjugated secondary antibody (1:100) at room temperature for 2 h. Nuclei were counter stained with DAPI (Invitrogen, San Francisco, CA, United States) for 10 min. Cells were viewed using high-content screening (IN CELL Analyzer 1000, GE, United States). The quantitative description of cytoplasm to nucleus translocation process of p38 was calculated by comparing the average pixel intensity of the cytoplasmic area around nucleus (RING) with its average pixel intensity inside the nuclear region (CIRC), i.e., “CIRC-RING Avg Inten Diff” calculated by software.

### Statistical Analysis

Data were analyzed using SPSS software version 21.0 (SPSS Inc., Chicago, IL, United States) and expressed as mean ± SD. Comparison of multiple groups was performed using one-way analysis of variance (ANOVA), followed by the Bonferroni *post hoc* test when equal variances assumed, or Dunnett’s T3 *post hoc* test when equal variances not assumed, or Student’s *t*-test for comparison of two groups. Statistical significance was set at *P* < 0.05.

## Results

### Effects of Tamarixinin A on DBA/1 Mice with CIA

To investigate the pharmacological effects of tamarixinin A on RA development, the CIA model in DBA/1 mice was employed. On day 30, as significant swelling of hind feet developed compared with the blank control, mice received different therapies. As shown in **Figure 2B**, there was a significant decrease in body weight after immunization, compared to blank controls. After treatment with 50 mg/kg tamarixinin A, this loss was significantly decreased, compared with the CIA model group (*P* < 0.01).

After immunization, the arthritis symptoms in the CIA mice dramatically progressed and maintained the highest intensity during day 30 to day 44. The representative photographs of the hind paws in each group on day 44 are shown in **Figure 2C**. There was significant inflammatory swelling and erythema in control paws, and this was obviously relieved after tamarixinin A treatment. From day 35, compared with CIA model group, the mean clinical scores were significantly reduced after MTX, tamarixinin A 12.5 or 50 mg/kg treatment (*P* < 0.05 or 0.01) (**Figure 2D**).

As shown in **Figures 2E,F**, histological examinations showed that the CIA mice exhibited apparent degeneration of joint structure and narrowed joint space in the hind paw ankle joints with massive in soft tissue, synovial lining layer and cartilage destruction, and pannus formation. However, compare with CIA model mice, in tamarixinin A - or MTX-treated mice, the degree of inflammatory cell infiltration, bone erosion and degradation were significantly improved (*P* < 0.05 or 0.01).

Cytokines, such as TNF-α and IL-1β, are often associated with the progression and severity of arthritis. In this study, the level of TNF-α (**Figure 2G**) and IL-1β (**Figure 2H**) in serum were significantly increased compared with blank control group (*P* < 0.01). After tamarixinin A treatment, levels of TNF-α and IL-1β were significantly decreased, similar to that of MTX treated group.

In the CIA model, immunization by heterologous chicken CII results in the production of murine CII-specific autoantibodies. Results showed that CIA mice had significantly elevated levels of anti-mouse CII antibodies (*P* < 0.01). After tamarixinin A treatment, concentrations of anti-mouse CII autoantibodies were significantly reduced (**Figure 2I**).

This data suggests that tamarixinin A treatment elicits, to a certain extent, a therapeutic effect against arthritis in CIA mice.

### Effects of Tamarixinin A on Wistar Rats with AIA

Due to the complexity of human RA, using only one animal model cannot fully reflect the effects of the tested compound. Another frequently used animal model, the rat AIA model, was employed to confirm the anti- arthritic effect of tamarixinin A. As shown in **Figure 3B**, compared to control group, AIA rats exhibited serious swelling and erythema in hind feet since day 22, and maintained serious arthritis status until day 34. Mean clinical scores fluctuated in a narrow range, which were significantly lower after tamarixinin A (12.5 mg/kg and 25 mg/kg) and MTX treatment.

Compared with control rats, both the primary swelling of right hind paws (with adjuvant injected) and the secondary swelling of left hind paws (contralateral hind paw) in AIA rats exhibited significantly increased ankle diameter (*P* < 0.01) from day 22. Compared with the AIA model group, the primary paw swelling was significantly relieved by tamarixinin A 12.5, 25 mg/kg, and MTX treatment after day 29 (**Figure 3C**), and the secondary paw swelling was significant relief by tamarixinin A 25 mg/kg and MTX treatment after day 33 (**Figure 3D**).

Histological results show that the ankle joints in AIA rats exhibited apparent massive inflammatory cell infiltration in soft tissue, synovial lining layer, and cartilage destruction, pannus formation, and narrowed joint space. Tamarixinin A or MTX treatment might restrict the histological development of AIA (*P* < 0.05 or 0.01), as indicated by semi-quantitative scores in synovium hyperplasia, cartilage degradation, bone erosion, and soft tissue inflammation (**Figures 3E,F**).

Additionally, the level of TNF-α, IL-6 and IL-1β in joint space flushing fluid were tested by ELISA. Compared with the AIA model group, the levels of IL-6 were significantly decreased with tamarixinin A 12.5 mg/kg and 25 mg/kg treatment(**Figure 3G**), and the levels of IL-1β were significantly decreased after all doses of tamarixinin A treatment (**Figure 3H**). However, tamarixinin A treatment had an insignificant effect on the level of TNF-α (data not shown).

### Effects of Tamarixinin A on the Expression of p38, Phosphorylation of p38 and p65 in AIA Rat

To develop RA, p38 and p65 play a critical role in regulating inflammatory signaling pathways. To investigate the potential mechanisms of anti- RA effects of tamarixinin A, the expression of p38, phosphorylation of p38 and p65 were investigated in ankle joints of AIA rats. Compared with the AIA model group, the expression of p65 was significantly decreased with tamarixinin A 12.5 mg/kg and 25 mg/kg treatment (**Figure 4B**). Compared with the AIA model group, the expression of p38 was significantly decreased with tamarixinin A 12.5 mg/kg and 25 mg/kg treatment (**Figure 4C**), and the expression of p-p38 was significantly decreased after all doses of tamarixinin A treatment (**Figure 4D**).

### Effects of Tamarixinin A on LPS-Induced NO and Pro-inflammatory Mediators Production, and iNOS Expression *In Vitro*

**FIGURE 5:**
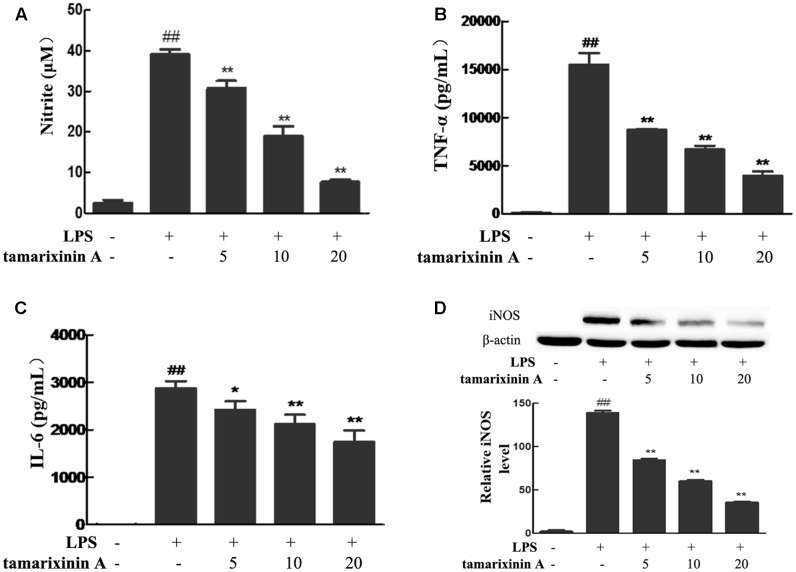
Effects of tamarixinin A on LPS-induced NO and pro-inflammatory mediators production, and iNOS expression *in vitro.* **(A)** The level of NO. **(B)** The level of TNF-α. **(C)** The level of IL-6. **(D)** iNOS expression in macrophages by western-blotting determined. Data are presented as means ± SD, *n* = 3. ^#^*P* < 0.05 and ^##^*P* < 0.01 versus untreated cells, ^∗^*P* < 0.05 and ^∗∗^*P* < 0.01 versus LPS-stimulated cells.

To confirm the anti- inflammatory effects *in vitro*, tamarixinin A was evaluated for its inhibition of NO and production of pro-inflammatory (TNF-α and IL-6) mediators in murine peritoneal macrophages stimulated by LPS. Compared with the LPS model group, the excessive NO (**Figure 5A**), TNF-α (**Figure 5B**) and IL-6 (**Figure 5C**) production was significantly inhibited (*P* < 0.05 or 0.01), and showed a dose-dependence.

Excessive NO in inflammatory diseases are produced through the inducible nitric oxide synthase (iNOS) pathway and amplify the inflammatory response. In this study, macrophages stimulated with LPS showed significant induction of iNOS expression, and as expected, the expression was suppressed upon treating the stimulated cells with tamarixinin A (**Figure 5D**).

### Effects of Tamarixinin A on the Phosphorylation of MAPK and NF-κB p65 Molecules in LPS Induced Macrophages

**FIGURE 6:**
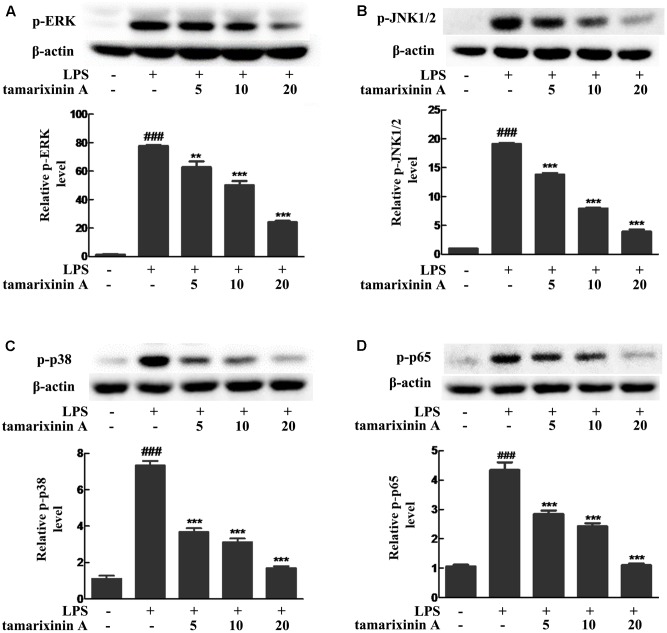
Effects of tamarixinin A on the phosphorylation of MAPK and NF-κB p65 molecules in LPS induced macrophages. The phosphorylation levels of ERK **(A)**, JNK1/2 **(B)**, p38 **(C)**, and p65 **(D)** were determined by Western blotting. Data are presented as means ± SD, *n* = 3. ^###^*P* < 0.001 versus untreated cells, ^∗∗^*P* < 0.01 and ^∗∗∗^*P* < 0.001 versus LPS-stimulated cells.

To develop RA, MAPK and NF-κB pathways play a critical role. To further investigate whether the anti- RA effects of tamarixinin A are associated with MAPK and NF-κB pathways inhibiton, phosphorylation of ERK, JNK1/2, p38, and p65 was investigated in LPS induced macrophages. As shown in **Figure 6**, LPS stimulation rapidly induced the phosphorylation of ERK, p38, JNK1/2 and p65 within 15 min in macrophages (*P* < 0.01), and tamarixinin A (5–20 μM) dose-dependently suppressed the phosphorylation of ERK (**Figure 6A**), JNK (**Figure 6B**), p38 (**Figure 6C**) and p65 (**Figure 6D**).

### Effects of Tamarixinin A on the Translocation of Cytosolic p38 into the Nucleus in LPS Induced Macrophages

**FIGURE 7:**
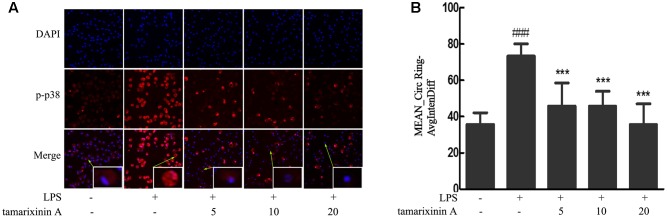
Effects of tamarixinin A on the translocation of cytosolic p38 into the nucleus in LPS induced macrophages. **(A)** LPS stimulation induced a nuclear translocation of p38 with red fluorescence and nucleus in blue fluorescence indicated by DAPI. **(B)** The capacity of p38 translocation from the cytosol to the nucleus is presented as values of Mean_CircRingAvgIntenDiff. Data are presented as means ± SD, *n* = 3. ^###^*P* < 0.001 versus untreated cells, ^∗∗∗^*P* < 0.001 versus LPS-stimulated cells.

Consequent to the above observations of p38 phosphorylation, nuclear translocation of p38 was investigated by high-content immunofluorescence screening. As shown in **Figure 7**, a significant nuclear translocation of p38 was induced with LPS stimulation which was significantly suppressed in a concentration - dependent manner (5–20 μM) after tamarixinin A treatment.

## Discussion

Rheumatoid arthritis is commonly characterized by progressive destruction of bone and cartilage, as well as intense and destructive infiltration of synovial tissue by a broad spectrum of inflammatory cells (Zhang et al., 2015). It is well known that each animal model cannot reflect the complexity of human disease. CIA in mice and AIA in rats are the two most common models for human RA and are used to evaluate and determine the effectiveness of novel therapeutic agents (McNamee et al., 2015). In general, the CIA model is widely used for disease pathogenesis and validation of potential therapeutic targets studies, while the AIA model is often used to investigate an experimental autoimmune disease with several features of human RA (Lorenzo et al., 2012). Based on data of approved drugs, using both models is more convincing for predicting clinical efficacy than using either model alone (Kannan et al., 2005; Hegen et al., 2008). Therefore, in this study, both CIA mice and AIA rat models were used to evaluate efficacy of tamarixinin A. The results show that tamarixinin A markedly suppresses inflammation and progression of arthritis in both animal models based on arthritis score, paw swelling, and thickness. Histopathological evaluations revealed that tamarixinin A significantly reduced joint destruction by inhibiting inflammation of soft tissue and synovium and preventing cartilage degradation and bone erosion. In addition, tamarixinin A might prevent body weight loss in CIA mice. It is well known that NSAIDs and MTX exert their therapeutic effects on RA through different pharmacological mechanisms; the major function of MTX is to prevent bone destruction, whereas NSAIDs have an anti-inflammatory effect. In this study, our data indicated that tamarixinin A 50 mg/kg treatment had better anti-inflammatory effect and anti-joint destruction effect in CIA mice. Tamarixinin A 25 mg/kg treatment had comparative anti-inflammatory effect in AIA rat. Overall, these results suggest that tamarixinin A has a significant therapeutic effect by suppressing inflammatory response, preventing joint destruction, and blocking the progression of arthritis in both CIA mice and AIA rats.

In RA, activated immune cells infiltrating the synovial tissue secrete large quantities of pro-inflammatory cytokines, as well as other cytokines and chemokines (Grabiec et al., 2012). Among pro-inflammatory cytokines, TNF-α is the master element of inflammation (McInnes and Schett, 2011), and IL-6 and IL-1β are downstream mediators of TNF-α (Zheng et al., 2014), which together play a critical role in mediating synovitis and joint destruction in RA (Xin et al., 2014). Among activated immune cells in RA, macrophages, which produce TNF-α, IL-6 and IL-1β (Zheng et al., 2014; Clavel et al., 2016) and tissue-degrading enzymes in the inflammatory infiltrate synovial sublining and at the pannus-cartilage interface (Davignon et al., 2013; Yeo et al., 2016), are central players in the pathogenesis (Szekanecz and Koch, 2007) and are an ideal target for the treatment of RA (Davignon et al., 2013). Biologicals blocking TNF-α, IL-6 and IL-1 are currently used in clinic with therapeutic potential (Smolen et al., 2014). Besides, the anti-inflammatory and analgesic mechanism for NSAIDs is partially due to their ability to inhibit the pro-inflammatory cytokines and iNOS-derived NO formation. In this study, tamarixinin A has therapeutic effects against RA by blocking the expression of TNF-α and IL-1β in the serum of CIA mice, and by blocking the expression of IL-6 and IL-1β in joint space flushing fluid of AIA rats. Tamarixinin A might also significantly inhibit TNF-α, and IL-6 secretion in LPS stimulated macrophages *in vitro*. Additionally, increased NO levels in serum and synovial fluid of RA patients generate reactive nitrogen species and reactive oxygen species and mediate the inflammation and joint destruction (Pal et al., 2015). Tamarixinin A also significantly inhibited the production of NO in LPS stimulated macrophages *in vitro.* Data also showed that the inhibitory effect of tamarixinin A on NO was associated with inhibition of iNOS expression. These results suggested that tamarixinin A could suppress inflammatory cytokine production/expression in macrophages and further reduce inflammation in RA.

Since some patients with RA remain non-responsive to anticytokine therapies (Smolen et al., 2014), there is growing interest in identifying novel therapeutic targets which could suppress inflammatory cytokine production. Among the potential targets, MAPKs and NF-κB have become important (IB, 2005; Brennan et al., 2006; Zhang et al., 2015), as they are involved in the transcriptional activation of a vast number of inflammatory and apoptotic machinery genes in response to TNF-α and suppress joint destruction (Zwerina et al., 2006; Olkkonen et al., 2015). Therefore, whether the inhibitory effect of tamarixinin A on proinflammatory cytokine production is associated with MAPK and NF-κB pathway activation was investigated. The *in vivo* results showed that the expression and phosphorylation of p38 and the expression of p65 were significantly downregulated after tamarixinin A treatment. *In vitro* results showed that phosphorylation of p38, ERK, JNK and p65 was triggered in macrophages by LPS stimulation which was suppressed by pretreatment of tamarixinin A. Furthermore, tamarixinin A pretreatment also significantly inhibited the translocation of p38 in LPS stimulated macrophages.

## Conclusion

These results provide evidence that tamarixinin A, a natural product isolated from *M. bracteata*, has a potential therapeutic effect on the development and progression of RA. Tamarixinin A suppresses the inflammatory response and prevents joint destruction, at least in part, through down-regulating MAPK and NF-κB signal pathway activation. Thus, there still a crucial need for tamarixinin A in RA therapy. Although that less toxic to murine macrophages has been shown previously, its side-effects *in vivo* should be further studied in the future. And also, besides MAPK and NF-κB signal pathway, the other pathways need to be studied.

## Author Contributions

ML, YZ, JB, and YD performed all the *in vivo* experiments in this study. HY, YF, and PM performed all the *in vitro* experiments in this study. JL and SL provided the compound in this study. ML and QH designed the experiments and wrote the paper.

## Conflict of Interest Statement

The authors declare that the research was conducted in the absence of any commercial or financial relationships that could be construed as a potential conflict of interest.
